# Mutation of brain aromatase disrupts spawning behavior and reproductive health in female zebrafish

**DOI:** 10.3389/fendo.2023.1225199

**Published:** 2023-06-23

**Authors:** Katherine Shaw, Mylène Therrien, Chunyu Lu, Xiaochun Liu, Vance L. Trudeau

**Affiliations:** ^1^ Department of Biology, University of Ottawa, Ottawa, ON, Canada; ^2^ State Key Laboratory of Biocontrol, Institute of Aquatic Economic Animals and Guangdong Province Key Laboratory for Aquatic Economic Animals, School of Life Sciences, Sun Yat-Sen University, Guangzhou, China

**Keywords:** aromatase, brain, *cyp19a1b*, estrogen, hormone, neuroendocrine, sexual behavior, zebrafish

## Abstract

Aromatase (Cyp19a1) is the steroidogenic enzyme that converts androgens into bioactive estrogens, and hence is in a pivotal position to mediate reproduction and sexual behavior. In teleosts, there are two aromatase paralogs: *cyp19a1a* that is highly expressed in granulosa and Leydig cells in the gonads with critical function in sexual differentiation of the ovary, and *cyp19a1b* that is highly expressed in radial glial cells in the brain with unknown roles in reproduction. *Cyp19a1* ^-/-^ mutant zebrafish lines were used to investigate the importance of the *cyp19a1* paralogs for spawning behavior and offspring survival and early development. Mutation of *cyp19a1b* was found to increase the latency to the first oviposition in females. Mutation of *cyp19a1b* in females also increased the number of eggs spawned; however, significantly more progeny died during early development resulting in no net increase in female fecundity. This finding suggests a higher metabolic cost of reproduction in *cyp19a1b* ^-/-^ mutant females. In males, the combined mutation of both *cyp19a1* paralogs resulted in significantly lower progeny survival rates, indicating a critical function of *cyp19a1* during early larval development. These data establish the specific importance of *cyp19a1b* for female spawning behavior and the importance of the *cyp19a1* paralogs for early larval survival.

## 1 Introduction

Androgens and estrogens are two major groups of sex steroids that play critical roles in vertebrates to coordinate the physiology and behavior of an individual with its environment. Aromatase (Cyp19a1) is the terminal steroidogenic enzyme that converts the aromatizable androgens, testosterone and androstenedione, into estradiol (E2) and estrone, respectively. In birds and most mammals, there is only one aromatase gene, *cyp19a1*, whose differential tissue distribution is attributed to differences in splicing of 5’ untranslated promoter regions across tissues (1). In contrast, teleosts possess two distinct *cyp19a1* genes, *cyp19a1a* and *cyp19a1b*, with differential tissue distribution. This is due to distinct regulatory elements in their promoter regions and differences in the presence of required transcription factors in specific cell types (2). In many teleosts, *cyp19a1a* is the most highly expressed aromatase in granulosa and Leydig cells in the gonads; while *cyp19a1b* is expressed at much higher levels in the brain, and specifically in radial glial cells (RGCs; 3).

The ovaries are a major source of estrogens that are released into systemic circulation to prepare the body for reproduction (4). Loss of aromatase expression significantly impairs female fertility and fecundity, at least in part, through loss of ovarian estrogen production. For example, total aromatase knockout (tAroKO) female mice, which lack whole body aromatase expression, are infertile due to disrupted follilculogenesis and ovulation failure (5). In teleosts, short term chemical inhibition of aromatase reduces female fertility and fecundity through impaired oocyte development and reduces plasma vitellogenin levels (6–8). Long term aromatase inhibitor treatment induces more dramatic effects such as ovarian retraction followed by testis formation resulting in female-to-male sex change (9, 10). Since chemical inhibitors are non-selective for the two Cyp19a1 isoforms, it only recently became possible to begin identifying their independent contributions to reproduction via the creation of zebrafish *cyp19a1* ^-/-^ mutant lines. It was discovered that *cyp19a1a* expression is critical for sexual differentiation of the ovaries while *cyp19a1b* expression is not required for this process (11–13). Though *cyp19a1a* is not required for testis differentiation, it is expressed at low levels and differences have been observed in the importance of its expression for male fertility in mice and teleosts. For example, the testis of male tAroKO mice were found to have arrested germ cell development at the spermatid stage as well as impaired sperm motility (14–17). In contrast, there were no developmental abnormalities observed in the testis of zebrafish *cyp19a1a* ^-/-^ mutants compared to wild-type (WT) males (18). Rather, the testis of *cyp19a1a* ^-/-^ mutants had more spermatozoa and higher levels of spermatogenesis-related genes, and these mutant males displayed normal fertility levels. These findings reveal critical roles of ovarian aromatase expression for female fertility and fecundity in mice and teleosts, whilst differences have been observed for testicular aromatase importance in male fertility, with *cyp19a1a* being dispensable for male teleost fertility.

A second major source of estrogens is the brain. In birds and mammals, the *cyp19a1* gene contains a brain-specific promoter region that specifies constitutive neuronal expression (19–22). Teleosts have a second paralog, *cyp19a1b*, that is expressed exclusively in RGCs due to the presence of G x RE, the glial x responsive element, in the promoter region (2). Increasing evidence has identified important roles for brain-derived estrogens in reproduction. For example, both female and male total tAroKO mice display reduced sexual behavior (23–26) and impaired olfactory discrimination (25, 27) that together suggests an involvement of brain estrogens in social recognition. The recent creation of a male brain aromatase knockout (bAroKO) mouse line identified the important role of brain-derived estrogens in male sexual behavior. Male bAroKO mice were found to have a significantly longer latency to the first mount event and a trend, though not significant, towards a greater latency to the first intromission event when paired with a hormonally primed female compared to WT males in sexual behavior assays (28). Female bAroKO mice have not yet been studied. In teleosts, studies have identified impairments in social recognition following chemical aromatase inhibition, such as reduced dominant male aggression in social behavior assays (29, 30). There has been comparatively less study, however, of effects on sexual behavior. This is likely due, at least in part, to the inability to identify specific Cyp19a1b- versus Cyp19a1a-induced effects on reproduction with the use of chemical aromatase inhibitors. There is strong evidence, however, to suggest that brain-derived estrogens likely play an important role in teleost sexual behaviors. Firstly, *cyp19a1b* is expressed in numerous brain regions important for sexual behavior (31). Secondly, *cyp19a1b* is a known estrogen-regulated gene due to the presence of an estrogen response element in its promoter region (32). This observation suggests that increased systemic estrogen levels, via high ovarian Cyp19a1a activity, likely drive increased *cyp19a1b* expression to prepare the brain for sexual behavior at the time in which the ovaries are prepared for reproduction.

Here we report that mutant *cyp19a1b* ^-/-^ females exhibit a longer latency to oviposition and release a significantly higher number of eggs during spawning compared to WT females. However, there was a higher larval mortality, resulting in no net fecundity differences between female *cyp19a1b* ^-/-^ mutant and WT pairings. A significantly higher larval mortality rate was found in progeny from *cyp19a1a* ^-/-^;*cyp19a1b* ^-/-^ mutant male pairings. These data reveal the importance of *cyp19a1b* for zebrafish reproduction.

## 2 Materials and methods

### 2.1 Experimental animals

Procedures used in this study were approved by the University of Ottawa Animal Care Committee and follow the guidelines of the Canadian Council on Animal Care for the use of animals in research. All fish were reared at the University of Ottawa Aquatics Facility according to standard housing procedures. The *cyp19a1* ^-/-^ mutant lines and WT zebrafish used in the experiments were all derived from a parental zebrafish AB strain to ensure identical genetic backgrounds amongst the groups for assessing the effects of *cyp19a1* mutation on spawning behavior. The *cyp19a1* ^-/-^ mutant lines were generated using the transcription activator-like effector nucleases (TALEN) genome editing system to create indel mutations at target sites in each of the *cyp19a1* paralogs producing frame-shift mutations (13). Mutation of *cyp19a1a* impairs sexual differentiation of the ovary resulting in all male populations of *cyp19a1a* ^-/-^ and *cyp19a1a* ^-/-^;*cyp19a1b* ^-/-^ mutant lines, i.e., only male mutants can be tested in these lines. These males have significantly lower serum E2 levels compared to WT males due to the contributions of gonadal aromatase expression to circulating estrogen levels. Mutation of *cyp19a1b* does not affect the sex ratio of the mutant line allowing testing of *cyp19a1b* ^-/-^ mutant effects on both males and females, and serum E2 levels in these fish are not significantly different from their WT counterparts. Fish were housed in 10-L tanks with dechloraminated water at 28°C and maintained on a 14:10 light-dark cycle and fed twice daily. All fish tested in this experiment were between the ages of 5-11 months post-fertilization and had no previous experience in the sexual behavior assay; with males and females separated into same-sex tanks at sexual maturation to prevent sexual interactions.

### 2.2 Behavioral tests

For all trials, mutant fish were size-matched within 2 mm body length (<10% body length difference) to an opposite sex WT fish. The evening before each experiment, paired fish were transferred to a 1-L testing tank containing an insert at the bottom for egg collection and a divider in the middle of the tank to keep the male and female separate before testing. The testing pair was allowed to acclimate overnight in a ZebraCube (Viewpoint Behavior Technology, Inc., Lyon, France) with the camera (either a Panasonic 16GB HC-V700M Full HD camcorder, Osaka, Japan or a Canon VIXIA HF R800 camcorder, Tokyo, Japan) present. The next morning, the pair was transferred to a new 1-L testing tank containing clean system water and the divider was removed and video recording started at 0900h (lights on). The fish pair was allowed to interact for 150 min, which was the most appropriate time determined in preliminary trials to capture the full timing of spawning behavior, particularly in the *cyp19a1b^-/-^* mutant lines. Videos were coded by date to ensure that the observer was blind to the treatment groups during viewing and video analysis using VLC media player (https://www.videolan.org/). Oviposition events, which represent spawning behavior, were selected as the most appropriate measure to identify changes in sexual behavior in this study. This decision was made based on the similar descriptive measures for the ethograms of sexual and aggressive behaviors in zebrafish (33, 34) that prevented unambiguous identification of motivational state during initial interactions in the video recordings. Since there is strong evidence identifying roles of brain-derived E2, and therefore, brain aromatase (i.e., Cyp19a1b), in both sexual and aggressive behaviors in vertebrates (29–31, 35), it was important to select a definitive measure of sexual behavior for analysis in this study. Oviposition events were characterized and were identified in the video recordings as the timing of gamete release during physical interaction. The oviposition times were manually recorded in an Excel spreadsheet for the later determination of the time to first and last spawning events, as well as the total number of spawning events in a trial.

### 2.3 Egg collection and eleutheroembryo rearing

Eggs were obtained from natural mating of a *cyp19a1* ^-/-^ mutant or WT fish paired with an opposite sex WT fish using the methods described above. Following the careful removal of the eggs from the bottom of the tank, they were then rinsed with clean system water and transferred to a Petri dish containing E3 medium at a density of 3 embryos/mL (36). At 4 h post-fertilization, unfertilized eggs were counted and removed from the Petri dish for the assessment of fertilization rates. All embryos were raised in an incubator at 28°C for the duration of the experiment. The following day (1-day post-fertilization; 1 dpf), any dead embryos were counted and recorded, then removed, following which E3 medium was replaced, and dishes were returned to the incubator. On 2, 3, and 4 dpf, any dead eggs were again counted and removed. Eleutheroembryos (i.e., hatched embryos) were counted and placed into separate Petri dishes containing E3 medium for measurements, and the medium was then replaced for the incubation of the remaining live eggs.

### 2.4 Zebrafish eleutheroembryo morphometrics

On day 2 post-fertilization, five eleutheroembryos were randomly selected from each clutch for measurement. The five measurements were later averaged to obtain a single value to represent the clutch at the given time. The eleutheroembryos were anesthetized with tricaine methanesulfonate (100 mg/L) and positioned under a dissecting microscope (Wild M10, Leica, Wetzlar, Germany) fitted with a phone mount and iPhone XS. Eleutheroembryos were gently positioned on their side to produce a lateral view for measurement of body length, eye area, and yolk sac area in photographs. A ruler was positioned under the microscope and photographed at the same magnification as used for Petri dishes to serve as a scale bar for image analysis. Following completion of the measurements, the eleutheroembryos were euthanized by immersion in an ice water bath.

### 2.5 Enzyme-linked immunosorbent assay

Female *cyp19a1b* ^-/-^ and WT fish were euthanized between 9h00 – 10h00 in an ice water bath, and brains and ovaries were immediately dissected, placed into individual labelled tubes, weighed, and tubes were then placed on ice. Homogenization buffer (90% methanol) was added to each tube, 150 µL per brain and 1000 µL per ovary sample, and samples were then sonicated for tissue dispersion and steroid release into solution. Samples were centrifuged (4°C, 13,200 rpm) for 10 min, and the supernatant was carefully removed and transferred to a new labelled tube. The samples were then evaporated to dryness (Labconco Centrivap Centrifugal Vacuum Concentrator, Model #7810014, 45°C, 1 h), and stored at 4°C overnight. The following day, 100 µL resuspension buffer (0.2% formic acid, 5% acetonitrile in water) was added to each sample tube, vortexed, and the tubes were then placed in a sonic water bath for 15 min to resuspend the samples. Following resuspension, the samples were run in C-18 solid-phase extraction columns (Catalog #r10.aq, Dr. Maisch HPLC GmbH, Ammerbuch-Entringen, Germany; >85% recovery rate in liquid chromatography tandem mass spectrometry) and the eluted samples were then evaporated to dryness (45°C, 3 h). The evaporated steroid residue was then resuspended in ELISA buffer, 200 µL for brain samples and 1000 µL for ovary samples, for 24 h at 4°C with intermittent vortexing prior to testing. Estradiol levels were measured using enzyme-linked immunoassay test kits (ELISA; Catalog #501890, Cayman Chemical, Ann Arbor, MI, USA) according to the manufacturer’s instructions. This assay has been extensively tested in teleost species (37–42) with an assay range of 0.61 – 10,000 pg/mL and sensitivity limit of 20 pg/mL. This E2 assay has very low levels of cross reactivity to other steroids such as cortisol, progesterone and testosterone (< 0.01%). All samples were run in duplicate on a single assay plate and only samples with intra-assay CVs < 10% were used in analyses.

### 2.6 Image analysis

**Figure 1:**
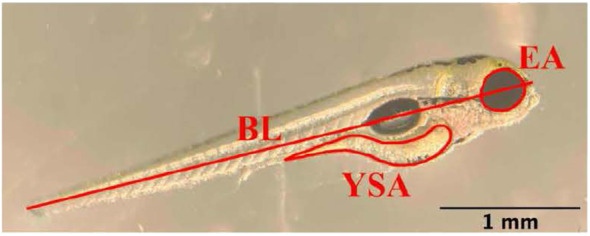
Depiction of the measurement of body length (BL), eye area (EA), and yolk sac area (YSA) in an eleutheroembryo on day 2 post-fertilization. Scale bar = 1 mm.

Photos were analyzed using ImageJ (v1.53) software. All images were analyzed by the same observer to ensure consistent measurements (i.e., to prevent inter-observer variability), and each measurement was repeated three times and the average value was calculated. Each image was measured for body length, eye area, and yolk sac area. Body length consisted of a straight-line measurement from the posterior tip of the notochord to the most anterior tip of the head passing through the eye (**Figure 1**). Eye and yolk sac areas were measured according to Martínez et al. (36) and traced with the freehand tracing tool in ImageJ (**Figure 1**).

### 2.7 Statistical analyses

Statistical analyses were conducted using GraphPad Prism v9 (GraphPad Software, Inc., La Jolla, CA, USA) with normality and homoscedasticity assessed using Shapiro-Wilk and Levene’s tests, respectively. For the behavior and eleutheroembryo analyses, normally distributed data were analyzed by either Student’s T test or One-Way ANOVA followed by Dunnett’s multiple comparisons tests for pairwise comparisons. Data that were not normally distributed were analyzed using either Mann-Whitney U or Kruskal-Wallis tests followed by Dunn’s multiple comparisons tests for pairwise comparisons. Data are presented as boxplots with the horizontal lines representing mean or median values, boxes representing interquartile ranges, and whiskers representing min-max values. For the E2 measurements, data were log-transformed and analyzed using a Two-Way ANOVA followed by Tukey’s multiple comparisons tests. For all data, significance is defined at p < 0.05 and all tests were assessed as two-tailed.

## 3 Results

### 3.1 Time to the first oviposition event

**Figure 2:**
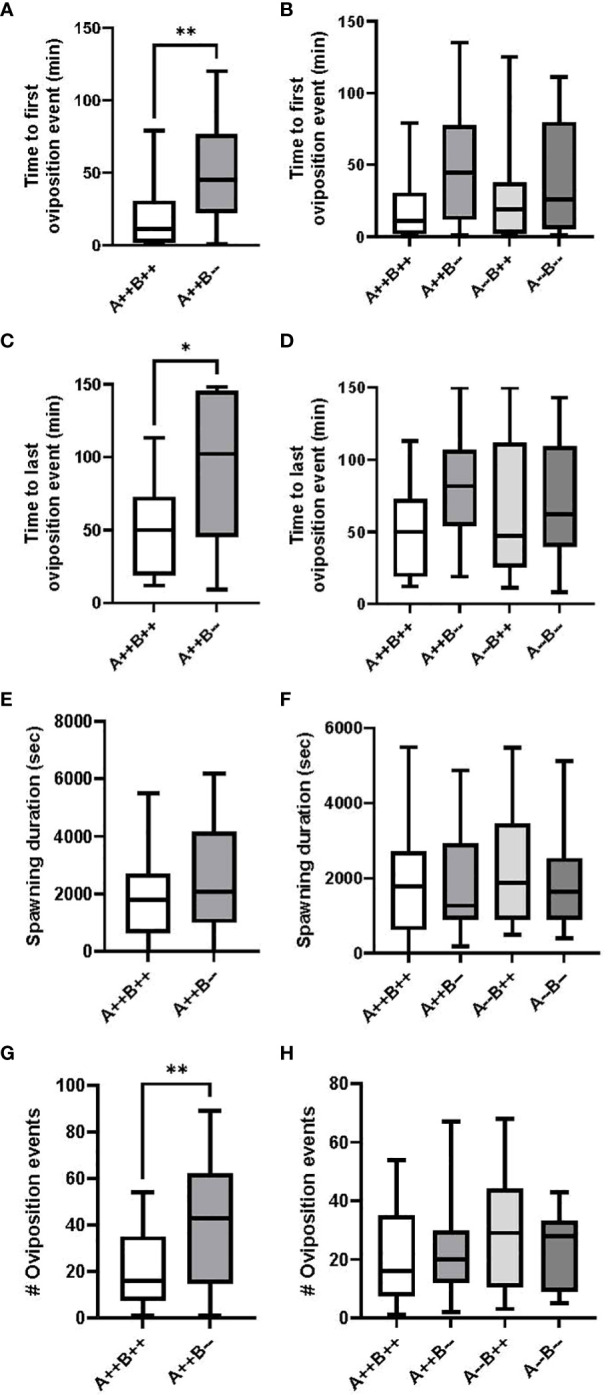
Timing and number of oviposition events during zebrafish pairwise mating trials. Test females (n=17 pairs) are displayed on the left **(A, C, E, G)** and test males (n=17-18 pairs) are displayed on the right **(B, D, F, H)**. Mann-Whitney U **(A, C, G)**, Student’s T **(E)**, Kruskal-Wallis **(B)** and One-Way ANOVA **(D, F, H)** tests were performed. Horizontal lines represent mean or median values, boxes represent interquartile ranges, and whiskers represent min-max values. Key to genotypes: A=*cyp19a1a*, B=*cyp19a1b*. Asterisks denote p<0.05 (*) and p<0.01 (**).

Female *cyp19a1b* ^-/-^ mutant fish took significantly more time to the first oviposition event compared to WT females (U(17,17)=70.50, p=0.0096; **Figure 2A**). On average, *cyp19a1b* ^-/-^ females took 4.1 times longer to the first oviposition event compared to WT females. There were no significant differences in the time to the first oviposition event among any of the male genotypes (H(3)=7.005, p=0.0717; **Figure 2B**).

### 3.2 Time to the last oviposition event

Female *cyp19a1b* ^-/-^ mutant fish took significantly more time to the last oviposition event compared to WT females (U(17,17)=81.50, p=0.0293; **Figure 2C**). On average, the last oviposition event in *cyp19a1b* ^-/-^ female pairings occurred 2 times later than in WT pairings. There were no significant differences in the time to the last oviposition event among any of the male genotypes (F(3)=1.657, p=0.1850; **Figure 2D**).

### 3.3 Spawning duration

There were no significant differences in the spawning duration between the *cyp19a1b* ^-/-^ mutant and WT female groups (T(32)=1.130, p=0.2668; **Figure 2E**) or between any of the mutant and WT male groups (F(3)=0.3184, p=0.8120; **Figure 2F**).

### 3.4 Number of oviposition events

Female *cyp19a1b* ^-/-^ mutant fish had significantly more oviposition events compared to WT females (U(17,17)=68, p=0.0074; **Figure 2G**). On average, *cyp19a1b* ^-/-^ mutant females exhibited 2.7 times more oviposition events than WT females. There were no significant differences in the number of oviposition events among any of the male genotypes (F(3)=0.7744, p=0.5125; **Figure 2H**).

### 3.5 Fecundity

**Figure 3:**
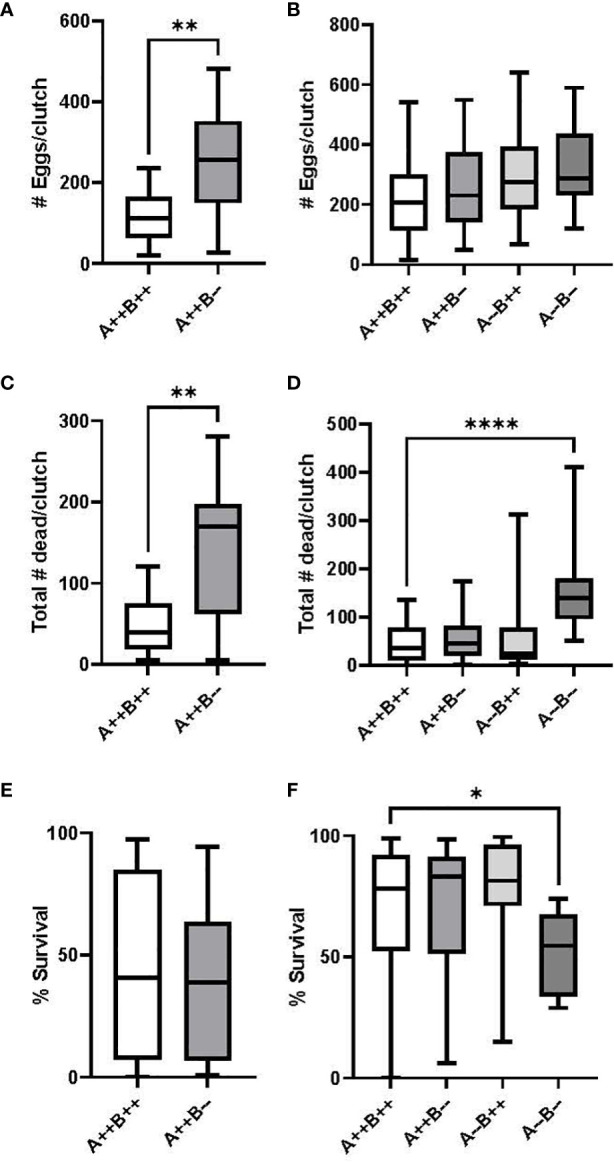
Number of eggs spawned per clutch and egg survival rate during zebrafish pairwise mating trials. Test females (n=14-16 pairs) are displayed on the left **(A, C, E)** and test males (n=16-23 pairs) are displayed on the right **(B, D, F)**. Mann-Whitney U **(A, C)**, Student’s T **(E)**, One-Way ANOVA **(B)** and Kruskal Wallis **(D, F)** tests were performed. Horizontal lines represent mean or median values, boxes represent interquartile ranges, and whiskers represent min-max values. Key to genotypes: A=*cyp19a1a*, B=*cyp19a1b*. Asterisks denote p<0.05 (*), p<0.01 (**), p<0.0001 (****).

There were no significant differences in fertilization rates between female *cyp19a1b* ^-/-^ mutant and WT female pairings (U(14,16)=90, p=0.3557, data not shown), with average fertilization rates of 99% and 93%, respectively. Female *cyp19a1b* ^-/-^ fish spawned significantly more eggs per clutch compared to WT females (U(14,17)=44, p=0.0022, **Figure 3A**); however, significantly more eleutheroembryos died by Day 4 from the female *cyp19a1b* ^-/-^ clutches compared to the WT clutches (U(14,16)=45, p=0.0043, **Figure 3C**), resulting in no net difference in fecundity between the female groups (T(28)=0.1303, p=0.8973, **Figure 3E**). On average, there were 2.3 times more eggs spawned by *cyp19a1b* ^-/-^ females and 4.2 times more progeny died by Day 4 from these mutant females compared to WT females.

Fertilization rates for WT, *cyp19a1b* ^-/-^, *cyp19a1a* ^-/-^, and *cyp19a1a* ^-/-^; *cyp19a1b* ^-/-^ group pairings were 99%, 98%, 95%, and 99%, respectively, with no significant differences observed among the male groups (H(3)=3.039, p=0.3856, data not shown). There were no significant differences in the number of eggs per clutch between any of the male group pairings (F(3)=1.649, p=0.1857, **Figure 3B**); however, there was a significant difference in the number of dead eleutheroembryos per clutch amongst the male genotypes (H(3)=23.91, p<0.0001). Pairwise comparisons revealed that significantly more progeny died by Day 4 from the male *cyp19a1a* ^-/-^; *cyp19a1b* ^-/-^ group pairings compared to the WT male pairings (p<0.0001, **Figure 3D**). There was also a significant difference in the survival rate of eleutheroembryos from the male group pairings (H(3)=14.13, p=0.0027). Pairwise comparisons revealed that there was a significantly lower survival rate in the double mutant male offspring compared to the WT male offspring (p=0.0203, **Figure 3F**). The survival rate of larvae from male *cyp19a1a* ^-/-^; *cyp19a1b* ^-/-^ pairings was 24% lower than WT pairings.

### 3.6 Eleutheroembryo morphometrics

**Figure 4:**
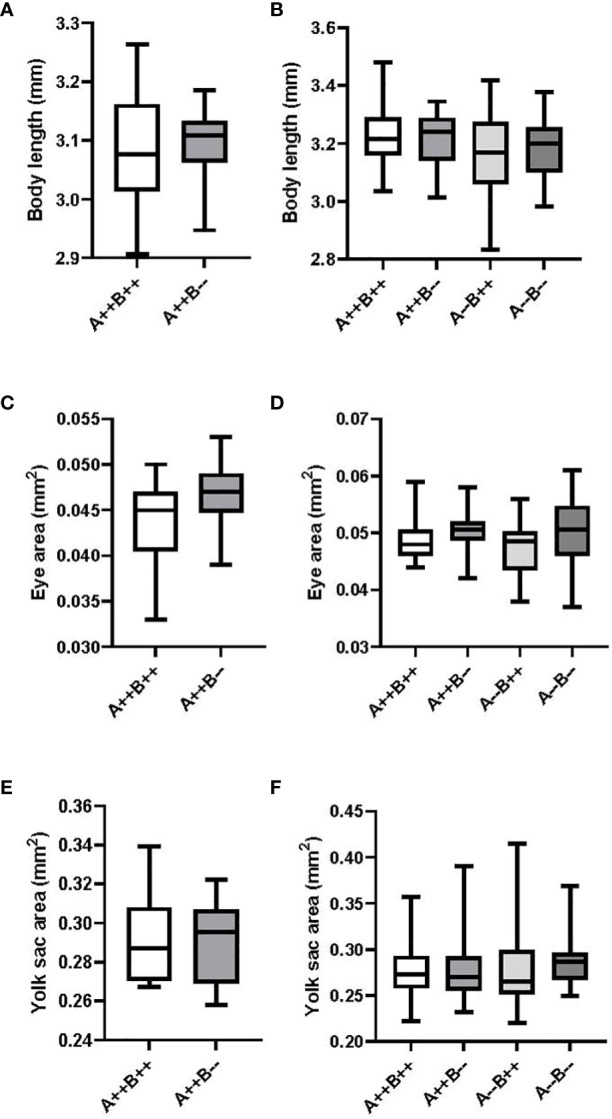
Body length, eye area, and yolk sac area measurements of eleutheroembryos from zebrafish pairwise mating trials on day 2 post-fertilization. Eleutheroembryos from test females (n=9-14) are displayed on the left **(A, C, E)** and from test males (n=16-21 pairs) are displayed on the right **(B, D, F)**. Student’s T **(A, C, E)**, One-Way ANOVA **(B)** and Kruskal-Wallis **(D, F)** tests were performed. Horizontal lines represent mean or median values, boxes represent interquartile ranges, and whiskers represent min-max values. No statistical differences between genotypes were evident. Key to genotypes: A=*cyp19a1a*, B=*cyp19a1b*.

There were no significant differences on day 2 between the body length (T(21)=0.1487, p=0.8832; **Figure 4A**), eye area (T(21)=1.647, p=0.1144; **Figure 4C**), or yolk sac area (T(21)= 0.09161, p=0.9279, **Figure 4E**) of offspring from female *cyp19a1b* ^-/-^ and WT females. There were also no significant differences on day 2 between the body length (F(3)=1.657, p=0.1844; **Figure 4B**), eye area (H(3)=5.172, p=0.1596, **Figure 4D**), or yolk sac area (H(3)=2.561, p=0.4644, **Figure 4F**) of offspring from any of the male genotypes.

### 3.7 Estradiol content in female brain and ovary

**Figure 5:**
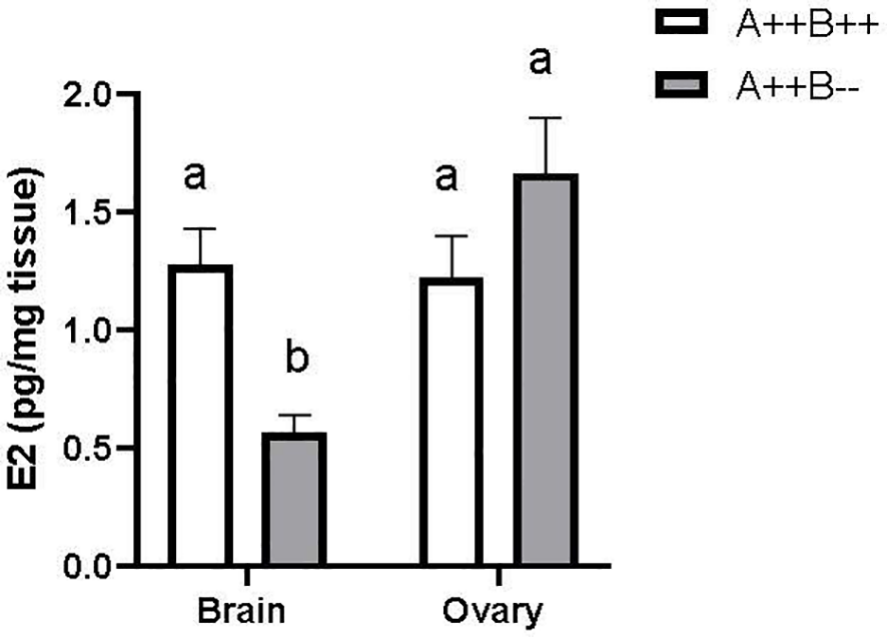
Estradiol (E2) levels in the brains and ovaries of adult WT (A++B++) and *cyp19a1b* ^-/-^ (A++B–) female zebrafish (n=5-8 per group). Data were log-transformed and analyzed using a Two-Way ANOVA followed by Tukey’s multiple comparisons tests. Data are plotted as means + SEM. Means with different letters a-b represent statistically significant differences (p<0.05). For clarity, the significant main effect of tissue type is not displayed. Key to genotypes: A=*cyp19a1a*, B=*cyp19a1b*.

There was a significant main effect of tissue type on E2 levels (F(1,21)=11.40, p=0.0029). Ovarian tissue had 1.6 times higher E2 levels compared to brain. There was no significant main effect of genotype on E2 levels (F(1,21)=3.295, p=0.0838). There was a significant tissue type X genotype interaction (F(1,21)=15.36, p=0.0008). The brains of *cyp19a1b* ^-/-^ mutant females had 2.3 times lower E2 levels compared to the brains of WT females (p=0.0066; **Figure 5**). The brains of *cyp19a1b* ^-/-^ mutant females also had 3 and 2.2 times lower E2 levels compared to their ovaries (p=0.0002) and to the ovaries of WT females (p=0.0080), respectively (**Figure 5**).

## 4 Discussion

This study is the first assessment of the independent contribution of *cyp19a1b* and by implication, brain estrogen production, to female spawning behavior in a teleost species. It was found that *cyp19a1b* ^-/-^ mutant female zebrafish exhibited an increased latency to initiate spawning and released higher numbers of eggs compared to WT females. These findings represent an important difference from the observed impairment of female fertility in chemical inhibitor studies in which both aromatase isoforms are inhibited in the body. For example, total aromatase inhibition reduced female fathead minnow (*Pimephales promelas*; 6), medaka (*Oryzias latipes*; 7) and zebrafish (8) fecundity. These differences are likely due to the contribution of circulating estrogens produced by the ovaries via Cyp19a1a that function to maintain the integrity of the ovarian state and the reproductive capacity (i.e., fertility) of female zebrafish (10). The observed effects on female spawning behavior in the current study are linked to altered local brain estrogen production via Cyp19a1b, because brain E2 levels are significantly lower in *cyp19a1b* ^-/-^ mutant females compared to WT females, whilst ovarian E2 levels are similar between the groups. The time to the last spawning event, but not the spawning duration, was also longer in the *cyp19a1b* ^-/-^ mutant females. This suggests that the mutation of *cyp19a1b* affects the perception of reproductive cues important for timely mate identification and assessment, since zebrafish rely heavily on visual and pheromonal cues for reproductive behavior (43). We note that *cyp19a1b* and estrogen receptors (*esr1*, *esr2a*, *esr2b*) are highly expressed at multiple levels of these sensory pathways including from the peripheral level of sensory nerve fibres to the levels of primary targets and sensory integration centres in the brain (31). Future study of *cyp19a1b* ^-/-^ mutant females will need to account for these potential multiple levels of sensory impairments as well as to identify downstream neuronal mediators of RGC-derived estrogens.

Our results resemble those observed in the tAroKO female mice that display reduced sexual behavior when paired with WT males (25), likely due to impaired brain estrogen signalling (44). There has been no study to date of specific bAroKO effects on female sexual behavior in mammalian models. However, male bAroKO male mice have impaired social recognition and a significantly longer latency to initiate mounting behavior with hormonally primed female mice compared to WT males (28). Our data for male zebrafish contrast those in mice as we observed no significant differences in spawning success between male *cyp19a1b* ^-/-^ mutants and WT fish. Rather, increasing evidence indicates that brain androgen signalling is critical for male teleost sexual behavior. It was recently discovered that *cyp17a1* ^-/-^ male zebrafish display reduced mating behaviors with WT females compared to WT males, which is likely a result of lower brain levels of testosterone and 11-ketotestosterone (45). The reduced contact time of *cyp17a1* ^-/-^ male mutants with WT females could be rescued following 11-ketotestosterone administration, indicating that non-aromatizable androgens regulate male sexual behavior (45). Evidence from androgen receptor gene editing studies also demonstrate the important role for androgen signalling in male teleost sexual behavior (46–48).

An important observation in the current study is that female *cyp19a1b* ^-/-^ mutants paired with WT males spawned significantly more eggs compared to WT females; however, there was a concomitant decrease in larval survival so that the total number of viable larvae produced was similar in female mutants and WT fish. One possible but unlikely explanation for the observed higher larval mortality is reduced circulating E2 and vitellogenin egg deposition in female *cyp19a1b* ^-/-^ mutants. Vitellogenin is a classical estrogen-regulated hepatic protein that nourishes the embryo during early development and is critical for embryo survival (49). Vitellogenin levels correlate with serum E2 and are significantly lower in females following administration of chemical aromatase inhibitors (6–8). The higher larval mortality in the current study is not due to changes in ovary-derived E2 in *cyp19a1b* ^-/-^ mutant females since the *cyp19a1b* ^-/-^ mutant and WT females have similar ovarian tissue (this study) and circulating E2 (13) levels. Moreover, there were no significant differences in yolk sac volume or body length at 2 dpf in progeny from *cyp19a1b* ^-/-^ mutant female and WT pairings. These two morphometrics are positively correlated in embryos and directly linked to maternal vitellogenin levels that are driven by systemic estrogens which bind to activate hepatic estrogen receptors (50, 51).

The *cyp19a1b* ^-/-^ mutant females released more than twice the number of eggs than WT females. This suggests that the mutation of Cyp19a1b may increase the energetic cost of reproduction due to the substantial metabolic investment in large quantities of larvae that do not survive to adulthood. It is well known that spawning is one of the most metabolically demanding activities for a fish (52). For example, the rate of gamete biomass production is roughly proportional to whole-organism metabolic rate, with female fishes allocating approximately half of their energy reserves towards reproductive function (53). It will be important for future studies to determine the contribution of Cyp19a1b to long-term fitness costs associated with higher reproductive investment by mutant females.

Higher mortality rates in larvae from *cyp19a1a* ^-/-^;*cyp19a1b* ^-/-^ pairings compared to WT pairings among the male genotypes also reveals a role for aromatase in males. While larval survival was similar between WT, *cyp19a1a* ^-/-^ and *cyp19a1b* ^-/-^ males, survival of offspring from the double mutant males was 24% lower than WT offspring. Thus, total aromatase activity of the father contributes to offspring survival. This idea is supported by previous studies reporting increased mortality in larval zebrafish during acute chemical aromatase inhibition (36, 54–57). Moreover, both *cyp19a1* transcripts are expressed during the first 48 h post-fertilization in zebrafish embryos, which suggests a role in early development and survival (58). While overall survival rate was lower in larvae from *cyp19a1a* ^-/-^;*cyp19a1b* ^-/-^ males, there were no obvious larval abnormalities observed and the size of the remaining survivors appeared relatively normal. Yolk sac area, eye area, and body length at 2 dpf were similar across all genotypes. These observations are similar to those of Gould et al. (59), reporting no effect of aromatase inhibition on larval zebrafish development. However, our study contrasts those demonstrating chemical inhibition of both aromatases affect one or multiple of these morphometrics (36, 56, 57, 60, 61). It is possible that significant differences in these morphometrics might emerge during later development after 2 dpf.

## 5 Conclusions

We have demonstrated a role of brain aromatase in female spawning behavior. Female zebrafish carrying a frameshift mutation in *cyp19a1b* had a longer latency to initiate spawning behavior and had higher numbers of eggs spawned compared to WT females. The importance of *cyp19a1b* for embryo survival was demonstrated by the increased mortality of progeny from female *cyp19a1b* ^-/-^ mutants and *cyp19a1a* ^-/-^;*cyp19a1b* ^-/-^ mutants compared to WT pairings. Further study will be needed to determine the downstream neuronal pathways through which brain estrogens produced in RGCs lead to the observed changes in female spawning behavior. It will also be important to determine the causes of increased mortality in eleutheroembryos from *cyp19a1b* ^-/-^ mutant females and *cyp19a1a* ^-/-^;*cyp19a1b* ^-/-^ mutant males.

## Data availability statement

The raw data supporting the conclusions of this article will be made available by the authors, without undue reservation.

## Ethics statement

The animal study was reviewed and approved by University of Ottawa Animal Care Committee.

## Author contributions

KS, MT, and VLT contributed to conception and design of the study. XL contributed the *cyp19a1^-/-^* mutant lines for study. KS and MT conducted data collection and performed statistical analyses. KS wrote the first draft of the manuscript. MT and CL wrote sections of the manuscript. All co-authors contributed to manuscript revision, read, and approved the submitted version.
